# Radioembolization (^90^Y) achieves higher response rates and reduces progression risk compared with DEB-TACE in hepatocellular carcinoma

**DOI:** 10.1097/HC9.0000000000000935

**Published:** 2026-04-17

**Authors:** Kelley Núñez, Navid Hasani, Julie Cronan, Juan Gimenez, Ari Cohen, Tyler Sandow, Paul Thevenot

**Affiliations:** 1 University of Queensland—Ochsner Clinical School, New Orleans, Louisiana, USA; 2 Institute of Translational Research, Ochsner Health System, New Orleans, Louisiana, USA; 3 Interventional Radiology, Ochsner Health System, New Orleans, Louisiana, USA; 4 Multi-Organ Transplant Institute, Ochsner Health System, New Orleans, Louisiana, USA; 5 Faculty of Medicine, University of Queensland, Brisbane, Queensland, Australia

**Keywords:** cirrhosis, liver disease, oncology, radiation, survival

## Abstract

**Background:**

Drug-eluting bead transarterial chemoembolization (DEB-TACE) and yttrium-90 (^90^Y) radioembolization are approved therapies to treat hepatocellular carcinoma (HCC). Several randomized controlled trials and propensity score-matched studies (PSM) have been conducted to compare these 2 treatments; many utilized ^90^Y standard dosimetry (<200 Gy), which produced inferior outcomes compared with modern-day ^90^Y personalized dosimetry, which yields tumor doses exceeding 205 Gy.

**Purpose:**

This study utilized PSM between DEB-TACE and ^90^Y with personalized dosimetry to compare treatment and patient outcomes in Barcelona Clinic Liver Cancer (BCLC) A–B HCC.

**Methods:**

This retrospective study included 258 patients with unresectable BCLC A–B stage HCC treated with DEB-TACE or ^90^Y as the initial treatment approach from 2015 to 2024. PSM was performed (^90^Y:DEB-TACE), matching for tumor burden and alpha-fetoprotein levels at diagnosis. The primary endpoint was target response rate with secondary endpoints of overall response, target retreatment rate (TTR), target and overall time-to-progression (TTP), and overall survival (OS).

**Results:**

Overall, ^90^Y achieved significantly higher target complete (CR) and objective response (OR) rates compared with DEB-TACE (71% vs. 33% and 88% vs. 58%), respectively. In multifocal disease, target CR rates were higher following ^90^Y (68% vs. 13%). ^90^Y also yielded a longer duration of CR with a 1-year target retreatment rate of 12% compared with 40% with DEB-TACE. This translated into a longer target TPP (*p*=0.030) with ^90^Y, although overall TPP and OS were similar between treatment modalities. In multifocal disease, ^90^Y generated superior response rates as well as target (*p*=0.007) and overall TTP (*p*=0.015).

**Conclusions:**

^90^Y with personalized dosimetry achieved higher response rates and extended the duration of complete responses compared with DEB-TACE. ^90^Y was also more effective at treating multifocal disease.

## Graphical abstract

**Figure:**
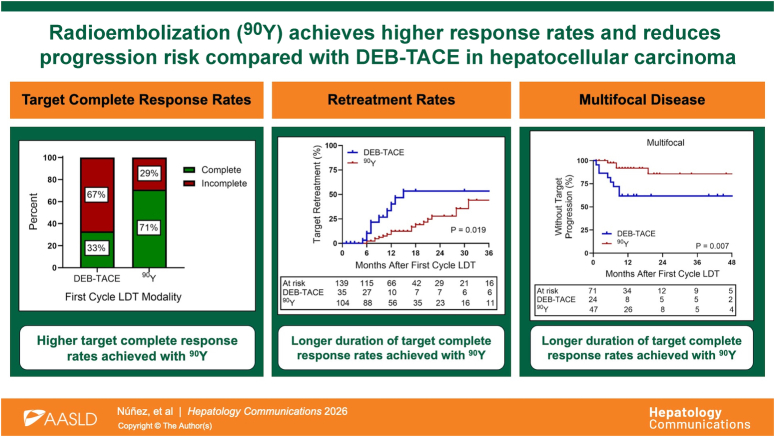

## INTRODUCTION

Hepatocellular carcinoma (HCC) is the sixth most diagnosed cancer and ranks third for cancer-related deaths.^1^,^2^ HCC incidence and mortality have also increased over the past 2 decades.^3^,^4^ As viral hepatitis-related HCC continues to decline, alcoholic liver disease (ALD) and metabolic dysfunction–associated steatotic liver disease (MASLD) are projected to emerge as the leading etiological drivers of HCC development.^1^ This growing burden underscores the need for effective therapies, particularly in HCC patients with barriers to surgical treatment (resection or liver transplantation), often limited to ideal surgical candidates with limited comorbidities, highly conserved liver function, and limited HCC burden.^5^

Liver-directed therapies (LDTs) play a vital role in managing early-stage to intermediate-stage HCC, where some modalities can serve as both definitive therapies and bridging/downstaging options to surgery. The most utilized LDTs for HCC >3 cm and multifocal disease include transarterial chemoembolization (TACE) and transarterial radioembolization (TARE). TACE is widely used to treat HCC across Barcelona Clinic Liver Cancer (BCLC) stages and includes either conventional (cTACE) or drug-eluting bead (DEB-TACE), with the latter demonstrating better objective response, disease control, and lower all-cause mortality rates compared with cTACE.^6^ TARE in the form of yttrium-90 (^90^Y) radioembolization has recently been incorporated into the BCLC algorithm^7^ with several trials demonstrating the ability to safely deliver ablative radiation doses using personalized dosimetry. This approach has yielded excellent first-cycle response rates in BCLC A–C disease.^8^,^9^,^10^

In clinical practice, the overall preference between TACE and ^90^Y, as well as how they may be applied across the BCLC continuum, can vary among institutions. To date, the field lacks consensus on the overall optimal transarterial approach as well as a consensus on whether either modality could provide superior outcomes in specific disease sub-stages (large solitary burden, unilobular versus multilobular, multifocal disease, or less compensated disease). The 2016 randomized phase II PREMIERE trial compared cTACE against ^90^Y using standard dosimetry. While the study did not reveal any difference in overall survival (OS), ^90^Y prolonged time-to-progression (TTP) compared with cTACE.^11^ Several attempts at retrospective propensity matching have been limited due to rapidly evolving technical approaches to ^90^Y, as well as the challenge of defining the cross-section of HCC patients ideally treated with transarterial therapy as an initial treatment approach.^12^,^13^,^14^,^15^ With ^90^Y recently emerging as the most utilized bridging therapy in the United States^16^ and consensus approaches to optimally employing TACE and TARE in HCC, the timing is ideal to compare modality-dependent outcomes in the context of current, contemporary clinical utilization. The dynamic change in treatment approach from a DEB-TACE–dominant algorithm to a ^90^Y-dominant algorithm was captured within a single-system, multi-center retrospective design to facilitate matching based on HCC burden and biomarkers across modality-restricted treatment eras.

## METHODS

### Study cohort and clinical variables

A retrospective, multicenter, single-system study was conducted and approved by the Ochsner Health System Institutional Review Board (#2019.308), was exempt from written consent, and followed the ethical guidelines set forth by the 1975 Declaration of Helsinki. All patients with a confirmed HCC diagnosis treated among 3 system interventional radiology programs between January 2015 and April 2024 were collected to evaluate study criteria. Study inclusion criteria were as follows: (i) unresectable HCC as determined by surgical oncologists in conjunction with the multidisciplinary tumor board, (ii) staged as BCLC A–B, (iii) Eastern Cooperative Oncology Group (ECOG) performance status of 0–1, (iv) selected to receive ^90^Y or DEB-TACE as a first-cycle approach to comprehensively treat HCC burden. Exclusion criteria were as follows: (i) concurrent malignancy, (ii) incomplete baseline/follow-up data or unclear prior treatment history, (iii) multicycle treatment plan consisting of mixed treatment modalities, (iv) BCLC—B with diffuse or extensive bilobular disease, and (v) Child–Pugh >B7. The assignment to ^90^Y versus TACE was not randomized and was based on multidisciplinary tumor board recommendations according to the center treatment algorithm at the time of review. Decisions to treat with transarterial therapy considered factors such as tumor size/location, vascular anatomy, and preference of the interventional radiologist.

Clinical variables were extracted from the electronic medical record and included general demographics, hepatology history, baseline serology including complete blood count and metabolic panels, and baseline oncology assessments of tumor burden and performance status. Liver disease severity was captured using Child–Pugh classification, Model for End-stage Liver Disease (MELD), and modified albumin–bilirubin (ALBI) scores. Tumor burden was characterized based on size burden to assess BCLC staging and alpha-fetoprotein (AFP) levels at the time of diagnosis.

### Propensity score model

The propensity score was developed by constructing a multivariate logistic regression with the prognostic factors of tumor burden (multifocal and solitary), tumor size, and AFP level as inputs and with treatment modality defined as the response. The probability values were transferred to the logit function to generate the identifier values for each patient in the cohort. The identifiers were sorted and rounded to the hundredths place value before matching.

### DEB-TACE procedure

Under moderate sedation, a selective hepatic arteriogram was performed, and tumor-feeding arteries were catheterized (segmental or lobar level, depending on tumor location). Drug-eluting embolic beads (100–300 μm microspheres loaded with doxorubicin 50–75 mg) were infused until stasis in the vessel was achieved. Embolization endpoints were standardized to near stasis.

### ^90^Y radioembolization with personalized dosimetry

Radioembolization was conducted using the 2-phase approach consisting of a mapping angiogram/dose planning session followed by a ^90^Y treatment session. Dosimetry used the Medical Internal Radiation Dose (MIRD) methodology, incorporating both the perfused volume and the lung shunt fraction from the mapping angiogram. Treatment was delivered via ^90^Y glass microspheres (TheraSphere; Boston Scientific) through segmental or subsegmental hepatic arteries. Treatment characteristics are summarized in Supplemental Table S1, http://links.lww.com/HC9/C301.

### First-cycle LDT response

Upon completion of the treatment cycle, tumor response was assessed at imaging follow-up using mRECIST criteria^17^ using triphasic CT or MRI, depending upon the initial staging imaging modality. A treatment cycle was defined as the treatment of the entire tumor burden with either DEB-TACE or ^90^Y. The target complete response (CR) rate was defined as a complete treatment response to the treated lesion per mRECIST. Objective response (OR) rate was defined as either a complete or partial response following the initial treatment per mRECIST. The tumor response was evaluated based on 3-month first-cycle follow-up imaging.

### Study endpoints

The primary study endpoint was the first-cycle LDT response. Secondary endpoints included target retreatment rate, non-target disease-free survival, target TTP (tTTP), overall TTP, and OS. Target retreatment rate was assessed in patients who achieved a target CR and defined as the time from the first cycle of ^90^Y or DEB-TACE until the patient required additional LDT to the previously treated lesion due to local recurrence. Non-target disease-free survival (ntDFS) was assessed in patients achieving a target CR and defined as new intrahepatic disease outside the initially targeted region of the liver. The interval was defined as the time from post–first-cycle ^90^Y or DEB-TACE imaging until new tumor development or until censoring conditions were met. tTTP was defined as the time from the first cycle of ^90^Y or DEB-TACE until progression to stage BCLC—C attributable to the initially targeted tumor. TTP was defined as the time from the first cycle ^90^Y or DEB-TACE until overall disease progression to stage BCLC—C. OS was defined as the time from the first cycle ^90^Y or DEB-TACE until death from any cause. For ntDFS, tTTP, and TTP, censoring was applied relative to the most recent follow-up imaging date and for any of the following conditions: (i) >6 months without surveillance or follow-up imaging, (ii) received a liver transplantation, (iii), all-cause mortality, or (iv) absence of the event of interest at the time of data analysis. For OS, censoring was applied at the time of last system contact for any patient (i) alive at the time of data analysis or (ii) without system contact for >1 year.

### Statistical methods

Data analysis was performed in JMP 17.0 (SAS Institute Inc., Cary, North Carolina), with graphical output generated using Prism 10.0 (GraphPad, La Jolla, California). Continuous variables were displayed as the median with interquartile ranges, while categorical variables were displayed as a percentage of the total. Univariate analysis was used to determine factors associated with first-cycle LDT modality. Kaplan–Meier survival curves of tTTP, TTP, ntDFS, target retreatment rates, and OS were generated in Prism and compared using log-rank tests with curves trimmed to the nearest 6-month time point when at-risk populations fell <10%.

## RESULTS

### Study cohort

The system population consisted of 1169 patients diagnosed with HCC initially treated with LDT. After applying study inclusion/exclusion criteria, 413 eligible patients remained who received a comprehensive first-cycle treatment approach using DEB-TACE or ^90^Y (Supplemental Figure S1, http://links.lww.com/HC9/C302). Propensity score matching (PSM) was performed based on tumor burden and AFP expression, resulting in a final cohort size of 258 patients with 108 receiving DEB-TACE and 150 receiving ^90^Y. Overall and LDT modality-stratified demographics are displayed in Supplemental Table S2, http://links.lww.com/HC9/C301. The overall cohort was primarily male (72%, 186/258) with hepatitis C virus etiology (51%, 131/258) and Child–Pugh A (85%, 220/258). All patients were BCLC A–B stage, with most (72%, 187/258) having solitary disease and 39% (100/258) having an AFP level >20 ng/mL. After PSM, most demographic factors were well controlled; however, differences were observed in age, cirrhosis etiology, albumin, and platelets (Supplemental Table S2, http://links.lww.com/HC9/C301). These factors were not incorporated into the PSM and did not impact progression rates or target CR rates (Supplemental Tables S3, S4, http://links.lww.com/HC9/C301).

### Modality-specific treatment response rates

All DEB-TACE and ^90^Y treatments were technically successful. Imaging response could be assessed in 98% (252/258) of the cohort with a median imaging follow-up of 57 days (IQR: 34–94 d) for ^90^Y and 34 days (IQR: 30–45 d) for DEB-TACE (Table 1). The initial treatment cycle target CR rate was 71% (104/146) with ^90^Y compared with 33% (35/106) with DEB-TACE (*p*<0.001). Similarly, the overall OR rate was 88% (128/146) with ^90^Y compared with 58% (61/106) with DEB-TACE (*p*<0.001) (Table 1).

**TABLE 1:** Treatment response rates based on first-cycle LDT modality

Demographic	Cohort	DEB-TACE	^90^Y	*p*
Follow-up imaging, days, median (IQR)	40 (31–76)	34 (30–45)	57 (34–94)	<0.001
Unable to assess response, n (%)	6 (2)	2 (2)	4 (3)	
Target complete response rate, n (%)				<0.001
Complete	139 (55)	35 (33)	104 (71)	
Incomplete	113 (45)	71 (67)	42 (29)	
Overall objective response rate, n (%)				<0.001
Objective	189 (75)	61 (58)	128 (88)	
Non-objective	63 (25)	45 (42)	18 (12)	

Abbreviations: ^90^Y, yttrium-90; DEE-TACE, drug-eluting bead transarterial chemoembolization; IQR, interquartile range; LDT, liver-directed therapy.

The higher response rates with ^90^Y may be driven by superior performance within specific subgroups within the prognostic factors utilized in the PSM. The PSM factor of tumor size was stratified to isolate small (≤3 cm) and intermediate to large (>3 cm) HCC, multifocal disease, and elevated AFP between treatment modalities to compare CR rates. ^90^Y had superior response rates in both small, solitary disease (^90^Y: 83%, 43/52 | DEB-TACE: 44%, 27/61) as well as solitary, intermediate to large disease (^90^Y: 62%, 29/47 | DEB-TACE 23%, 5/22) (Table 2). Higher CR rates with ^90^Y were even more pronounced in multifocal disease (^90^Y: 68%, 32/47 | DEB-TACE: 13%, 3/23) and those with elevated AFP at diagnosis (^90^Y: 74%, 37/50 | DEB-TACE: 28%, 13/47). This confirms that the higher CR response rates with ^90^Y were uniform across the spectrum of HCC burden and patients with elevated AFP expression at diagnosis.

**TABLE 2:** Target complete response rates based on propensity-matched characteristics

Groupings	Cohort	DEB-TACE	^90^Y	*p*
Solitary, lesion size, ≤3 cm, n (% of total)				<0.001
Target complete response	70 (62)	27 (44)	43 (83)	
Target incomplete response	43 (38)	34 (56)	9 (17)	
Solitary, lesion size, >3 cm, n (% of total)				0.002
Target complete response	34 (49)	5 (23)	29 (62)	
Target Incomplete Response	35 (51)	17 (77)	18 (38)	
Multifocal disease, n (% of total)				<0.001
Target complete response	35 (50)	3 (13)	32 (68)	
Target incomplete response	35 (50)	20 (87)	15 (32)	
AFP >20 ng/mL, n (%)				<0.001
Target complete response	50 (52)	13 (28)	37 (74)	
Target incomplete response	47 (48)	34 (72)	13 (26)	

Abbreviations: ^90^Y, yttrium-90; AFP, alpha-fetoprotein; DEE-TACE, drug-eluting bead transarterial chemoembolization.

### Modality-specific outcome characteristics in initial CR and incomplete responders

**FIGURE 1:**
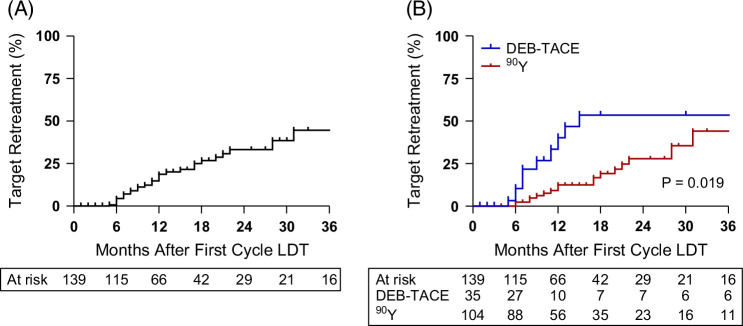
Target retreatment rates following the first cycle DEB-TACE or ^90^Y. (A) Overall target retreatment rate in patients who achieved a target complete response following the first cycle of DEB-TACE or ^90^Y. (B) Target retreatment rate in patients who achieved a target complete response following the first cycle of DEB-TACE or ^90^Y. Abbreviations: DEB-TACE, drug-eluting bead transarterial chemoembolization; LDT, liver-directed therapy; ^90^Y, yttrium-90.

While higher CR rates when treating with ^90^Y with personalized dosimetry at triphasic follow-up imaging or postsurgical have been well described, the critical outcome measure is whether these CRs translate into durable local disease control. Regardless of modality, patients achieving a target CR at imaging follow-up had a median time to target retreatment of 43 months (CI: 28–not reached), with 6-month and 1-yr retreatment rates of 4% and 19%, respectively (Figure 1A). The median time to target retreatment after a confirmed CR with ^90^Y was 43 months compared with 15 months with DEB-TACE. Target retreatment rates at 6 months, 1 year, and 2 years for ^90^Y were 2%, 12%, and 28% compared with 10%, 40%, and 53% for DEB-TACE (Figure 1B), confirming superior target disease control (*p*=0.019). The higher target CR and CR duration outcomes in ^90^Y did not influence future de novo HCC development, where new non-target disease development (^90^Y: 45% | DEB-TACE: 40%, *p*=0.591) and time to new disease development (^90^Y: median 6.8 mo | DEB-TACE: 6.5 mo, *p*=0.380) were similar to DEB-TACE complete responders (Table 3 and Supplemental Figure S2, http://links.lww.com/HC9/C302).

**TABLE 3:** Development of non-target HCC after target CR

	Cohort	DEB-TACE	^90^Y	*p*
No. of patients with target CR, n, % of total	139	35	104	
Development of de novo HCC, n, % of total				0.591
Yes	61 (44)	14 (40)	47 (45)	
No	78 (56)	21 (60)	57 (55)	
No. of LDT treatments until stage progression, median (IQR)	1 (1–2)	2 (1–4)	1 (1–2)	0.164
Switched modality, yes, n, % of total	24 (50)	6 (50)	18 (50)	1.00
AFP at time of de novo HCC, median (IQR)	11 (4.5–44)	8.3 (4.2–32)	11 (4.6–59)	0.552

Abbreviations: ^90^Y, yttrium-90; AFP, alpha-fetoprotein; CR, complete response; DEB-TACE, drug-eluting bead transarterial chemoembolization; HCC, hepatocellular carcinoma; IQR, interquartile range; LDT, liver-directed therapy.

In patients with an initial incomplete response, treatment outcomes were longitudinally monitored until achieving a target CR or overall progression to advanced-stage disease (BCLC—C). While the percentage of initial incomplete responders was higher with DEB-TACE, the percentage of incomplete responders who were successfully sequentially treated to CR was similar between modalities (^90^Y: 67% | DEB-TACE: 62%, *p*=0.615) (Table 4). Further, both the time to CR (6 mo) and the number of sequential treatments to CR (2 cycles) were similar between modalities (*p*=0.949).

**TABLE 4:** Initial incomplete responders treated to target complete response

	DEB-TACE	^90^Y	*p*
No. of patients with IC response, n, % of total	71 (67)	42 (29)	
Eventual target CR, n (% of IC responders)			0.615
Yes	44 (62)	28 (67)	
No	27 (38)	14 (33)	
No. of LDT to target CR, median (IQR)	2 (2–3)	2 (2–3)	0.949
Time until target CR, months, median (IQR)	6 (5–9)	6 (5–8)	0.949
Single modality type	24 (57)	16 (57)	
Switched modality	18 (43)	12 (43)	
No. of imaging appointments to target CR, median (IQR)	3 (3–4)	3 (3–4)	0.954

Abbreviations: ^90^Y, yttrium-90; CR, complete response; DEB-TACE, drug-eluting bead transarterial chemoembolization; IC, incomplete; IQR, interquartile range; LDT, liver-directed therapy.

### Modality-dependent tumor-restricted and overall outcomes

**FIGURE 2:**
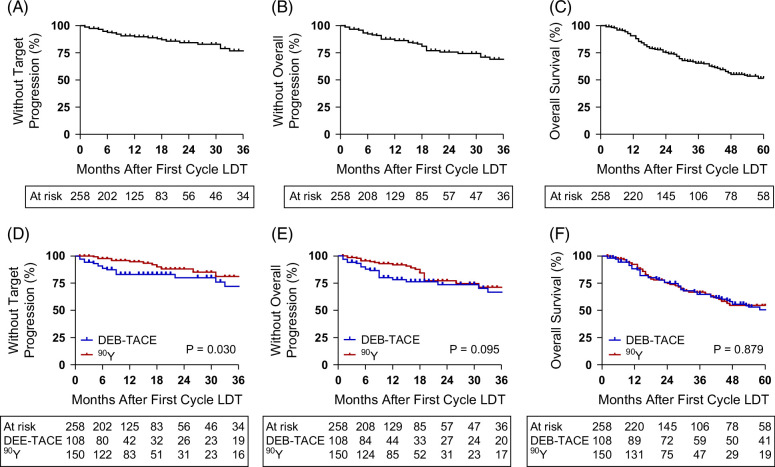
Overall outcomes for propensity-matched cohort. (A) Target time-to-progression, (B) overall time-to-progression, and (C) overall survival for the entire cohort. (D) Overall survival, (E) target time-to-progression, and (F) overall time-to-progression based on first-cycle modality. Abbreviations: ^90^Y, yttrium-90; DEB-TACE, drug-eluting bead transarterial chemoembolization; LDT, liver-directed therapy.

The median follow-up time for the cohort was 28 months, with a longer follow-up for patients in the DEB-TACE era (44 mo, IQR: 14–83 mo) compared with the more recent ^90^Y era (24 mo, IQR: 15–41 mo). The cohort median tTTP and overall TTP were not reached (Figures 2A, B), which yielded a median OS of 63 months (Figure 2C), and in agreement with BCLC OS projections for this cohort entirely consisting of BCLC—A and BCLC—B within downstaging criteria. While initial treatment modality had no effect on OS (*p*=0.879, Figure 2D), tTTP was inferior in the DEB-TACE cohort (*p*=0.030, Figure 2E) but did not translate to a difference when considering overall TTP (*p*=0.095, Figure 2F).

**FIGURE 3:**
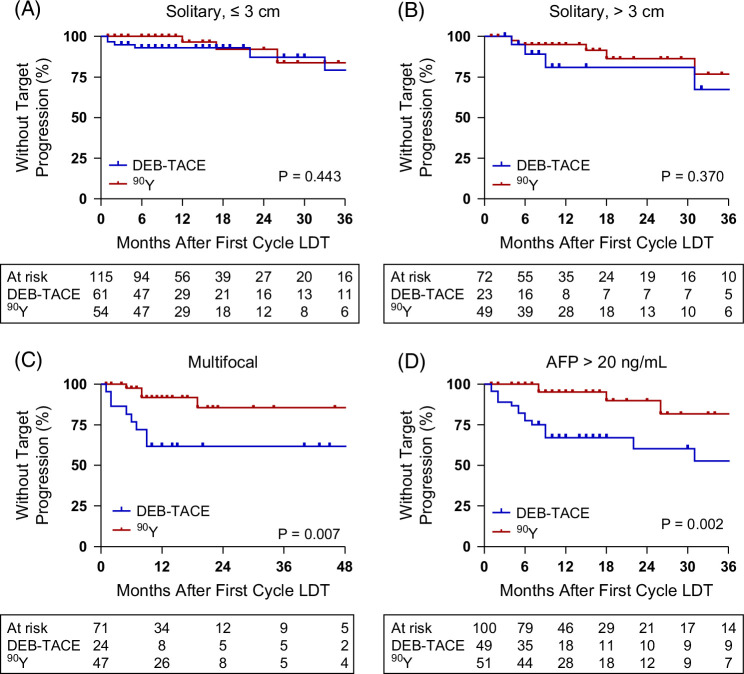
Target time-to-progression rates following first-cycle DEB-TACE or ^90^Y based on PSM. Target TTP following first-cycle LDT by modality in patients with (A) solitary lesion ≤3 cm, (B) solitary lesion >3 cm, (C) multifocal HCC, and (D) AFP levels >20 ng/mL at the time of diagnosis. Abbreviations: ^90^Y, yttrium-90; AFP, alpha-fetoprotein; DEB-TACE, drug-eluting bead transarterial chemoembolization; HCC, hepatocellular carcinoma; LDT, liver-directed therapy; PSM, propensity score matching; TTP, time-to-progression.

The notable difference in tTTP and overall TTP curve slope associated with a rapid post-LDT progression risk in DEB-TACE compared with ^90^Y could indicate superior performance within a specific PSM factor condition. This was investigated by comparing tTTP across subgroups within each PSM factor (Figure 3). Although ^90^Y had a higher CR rate in both small solitary (≤3 cm) and intermediate-large (>3 cm) disease, this did not translate into a tTTP benefit based on initial treatment modality (Figures 3A, B). However, tTTP was significantly improved in multifocal disease and those with elevated AFP compared with DEB-TACE (Figures 3C, D), although median tTTP was not reached with either modality. The 1-year target progression rates were 30% lower in multifocal disease (*p*=0.007) and 28% lower in elevated AFP (*p*=0.002) when treated with the first cycle of ^90^Y. The effect of ^90^Y also extended to a reduced overall risk of disease progression in both multifocal disease and those with elevated AFP (Supplemental Figure S3, http://links.lww.com/HC9/C302), with 1-year progression rate reductions of 41% in multifocal disease (*p*=0.015) and 30% in elevated AFP (*p*=0.006) but not with OS (Supplemental Figure S4, http://links.lww.com/HC9/C302).

## DISCUSSION

Currently, both DEB-TACE and ^90^Y are approved approaches to treat BCLC A–B HCC, with both modalities incorporated in the BCLC algorithm.^5^ However, modality selection remains at the discretion of providers with ^90^Y recently emerging as the bridge to transplant modality of choice in United States beyond the recommendations of the BCLC.^16^ While several randomized control trials^11^,^18^,^19^ and PSM studies^12^,^13^,^14^,^15^ have been performed for direct head-to-head comparisons between TACE and ^90^Y, many pre-date advancements in TARE personalized dosimetry^20^,^21^ or utilize sub-ablative radioembolization doses (<200 Gy). In this PSM study of nonresectable BCLC A–B, ^90^Y patients with personalized dosimetry achieved superior response rates, reduced retreatment rates, and extended tTTP compared with DEB-TACE.

Prior PSM studies comparing TACE with ^90^Y have used matching factors that are not associated with tumor-specific outcomes (age, cirrhosis etiology, ALBI grade), a narrowly focused treatment population (small, solitary disease ≤3 cm), mixed conventional with drug-eluting TACE, or utilized sub-ablative ^90^Y dosing.^12^,^13^,^14^,^15^ In contrast, this study focused on tumor burden and AFP levels before treatment to match parameters that directly impact LDT response rates and outcomes^22^,^23^ while utilizing modern clinical protocols for DEB-TACE and ^90^Y with personalized dosimetry, achieving high doses of >450 Gy. While age, cirrhosis etiology, albumin, and platelets were different between DEB-TACE and ^90^Y subgroups, these factors were confirmed not to be associated with CR rate or progression risk when controlled for treatment modality. The differences in cirrhosis etiology reflect the era-dependent differences in dominant HCC etiology from the HCV dominant era in DEB-TACE (2016–2020) compared with the MASLD/MASH dominant era in ^90^Y (2020–2024).

In nonresectable BCLC A–B disease, ^90^Y with personalized dosimetry led to higher target CR (71% vs. 33%) and overall OR (88% vs. 58%) rates compared with DEB-TACE, and this difference was across matching factors (multifocal disease and elevated AFP) and across small and intermediate-large, solitary disease. These results were comparable to other PSM studies comparing DEB-TACE versus ^90^Y, in which each study demonstrated higher target CR (87.3%–92.4%) and overall OR rates (57.4%–95%) for patients treated with ^90^Y compared with DEB-TACE (target CR: 49.1%–58.4%; overall OR: 52.6%–84.4%).^12^,^13^ In the TRACE trial, which randomized patients to either DEB-TACE or ^90^Y, overall OR rates for DEB-TACE were much higher (87%)^19^ than observed here (58%). However, it is important to note that the TRACE trial allowed for up to 5 sessions of DEB-TACE, and whether the sessions were preplanned or a CR was eventually obtained is unclear. Similar overall OR rates for ^90^Y were observed between the TRACE trial and this study (88% vs. 88%), respectively.^19^

Beyond the initial response rates reported in prior PSM studies and the TRACE trial, this analysis specifically focused on the duration of complete response as a direct measure of durable disease control. Notably, the duration of complete response has additional implications favoring ^90^Y over DEB-TACE related to the burden of multicycle treatments and the potential increased frequency of triphasic imaging encounters. The need for target retreatment was significantly higher after a completed DEB-TACE treatment cycle, resulting in a target CR, with 50% of patients requiring target retreatment at 15 months. This is in stark contrast to patients treated with ^90^Y, in which 15 months after a target CR, only 12% required retreatment of the primary tumor. Notably, these differences were not influenced by the development of non-target disease, which was equivalent regardless of the initial treatment approach. Collectively, this confirms prior data, which suggests a longer-lasting, durable tumor response in patients responding to ^90^Y. Considering the discordance rate between presurgical mRECIST and postsurgical pathology^24^ and the high complete pathologic necrosis rates observed with ^90^Y,^25^ the emerging data continue to suggest the likelihood of complete necrosis in the setting of a short-interval, imaging-based CR is superior with ^90^Y compared with DEB-TACE. The durable CR response rates with ^90^Y in this study also provide confirmation of prior results, which showed ^90^Y outcomes approaching those of patients undergoing curative treatments.^26^ Ablative ^90^Y dosing may provide a more effective strategy for completely treating HCC to the tumor margins, thereby decreasing the risk of developing local disease.

While initial non-CR rates were higher in the DEB-TACE group, many of these patients (44/71, 62%) were able to achieve a target CR with additional target treatment, with a similar bridge to CR rate as that observed for initial non-CR with ^90^Y (28/42, 67%). Despite more patients matched in the ^90^Y arm, the number of patients never achieving a target CR in the DEB-TACE group (27/108, 25%) was more than double that of the ^90^Y group (14/150, 9%). Our understanding of the changes in biological aggressiveness after incomplete LDT, or dependent upon the LDT mechanism employed (thermal, chemotherapy, radiotherapy), is limited. There is a large body of literature related to TACE refractiveness (see review^27^) compared with refractiveness to thermal or radiotherapy, and highlights a critical knowledge gap that may have important implications with increasing utilization of combination systemic therapy.

Despite differences in initial CR and duration of CR, there was no overall TTP or OS benefit associated with initial modality choice, in agreement with other comparative studies in the BCLC A–B population using ^90^Y with standardized dosimetry.^12^,^18^ However, a deeper investigation into the differences in tTTP between ^90^Y and DEB-TACE revealed significant differences in tTTP in multifocal disease as well as those with an elevated AFP >20 ng/mL. These target effects also translated into a reduction in overall TTP risk. A multifocal disease treatment responder effect for ^90^Y could be a critical development in bridge (BCLC—A) and downstage (BCLC—B) to liver transplantation, where a higher, more durable CR rate and improved disease control could expand access to potentially curative therapy. Regarding HCC biomarkers, research continues to show a direct correlation between complex biomarker expression and HCC biological aggressiveness and progression risk.^28^,^29^ An improved ability to treat aggressive disease could have immense implications in personalizing therapeutic approaches to improve HCC outcomes.

Several cost-effectiveness analyses across multiple institutions and in different countries have demonstrated that ^90^Y provides a cheaper option while improving quality of life.^30^,^31^,^32^,^33^ Cost analyses have consistently shown a long-term cost benefit in treating with ^90^Y over DEB-TACE even though the comparative cost of the procedure is higher.^31^ This PSM analysis identifies the critical outcomes behind the cost analysis through confirming a higher CR rate (reduced need for initial multicycle treatments) and a more durable CR (reduced target retreatment rate) while also highlighting that the cost effectiveness, quality of life, and overall outcomes may be even greater in multifocal and elevated AFP disease. The potential for ^90^Y cost-effectiveness in transplant downstaging compared with DEB-TACE has been highlighted in another recent study.^33^

Studies that compare LDT modalities retrospectively have challenges and limitations. The selection of initial treatment modality is complex and influenced by several factors, which can vary from center to center. In the current clinical landscape, centers that utilize both DEB-TACE and ^90^Y may have specific treatment algorithms or preferred strategies to sequence treatments, which cannot be effectively controlled without a multicenter trial employing a unified treatment algorithm. This propensity-matched study was possible due to a change in the center LDT algorithm, which isolated discrete DEB-TACE and ^90^Y treatment eras. However, this could potentially introduce a difference in the collective expertise of the provider team, as the collective experience of the tumor board and treatment providers may positively influence outcomes over time. Further, other unidentified or confounding effects related to underlying HCC etiology, improvements in HCC surveillance, and care access delivery could influence outcomes over time. In addition, due to the difference in modality application period, the study has a disparity in overall follow-up time, although this is unlikely to have influenced the short-term outcomes reported in the study.

In conclusion, the present PSM study demonstrated improved CR rates, extended duration of CR, and extended time to target disease progression in patients treated with ^90^Y with personalized dosimetry compared with DEB-TACE. Although neither modality yielded an overall difference in OS outcomes, CR rates, and progression risk in multifocal disease and AFP-elevated disease were improved and could have important implications in developing personalized approaches to LDT in HCC.

## Supplementary Material
