# Supplementary material for: Radioembolization (90Y) achieves higher response rates and reduces progression risk compared with DEB-TACE in hepatocellular carcinoma
Source: Hepatol Commun. 2026 Apr 17;10(5):e0935. doi: 10.1097/HC9.0000000000000935 (PMC13090074; doi:10.1097/HC9.0000000000000935)
Supplement: Supplementary file 1 [file hc9-10-e0935-s001.docx]

**SUPPLEMENTAL FIGURES**

**
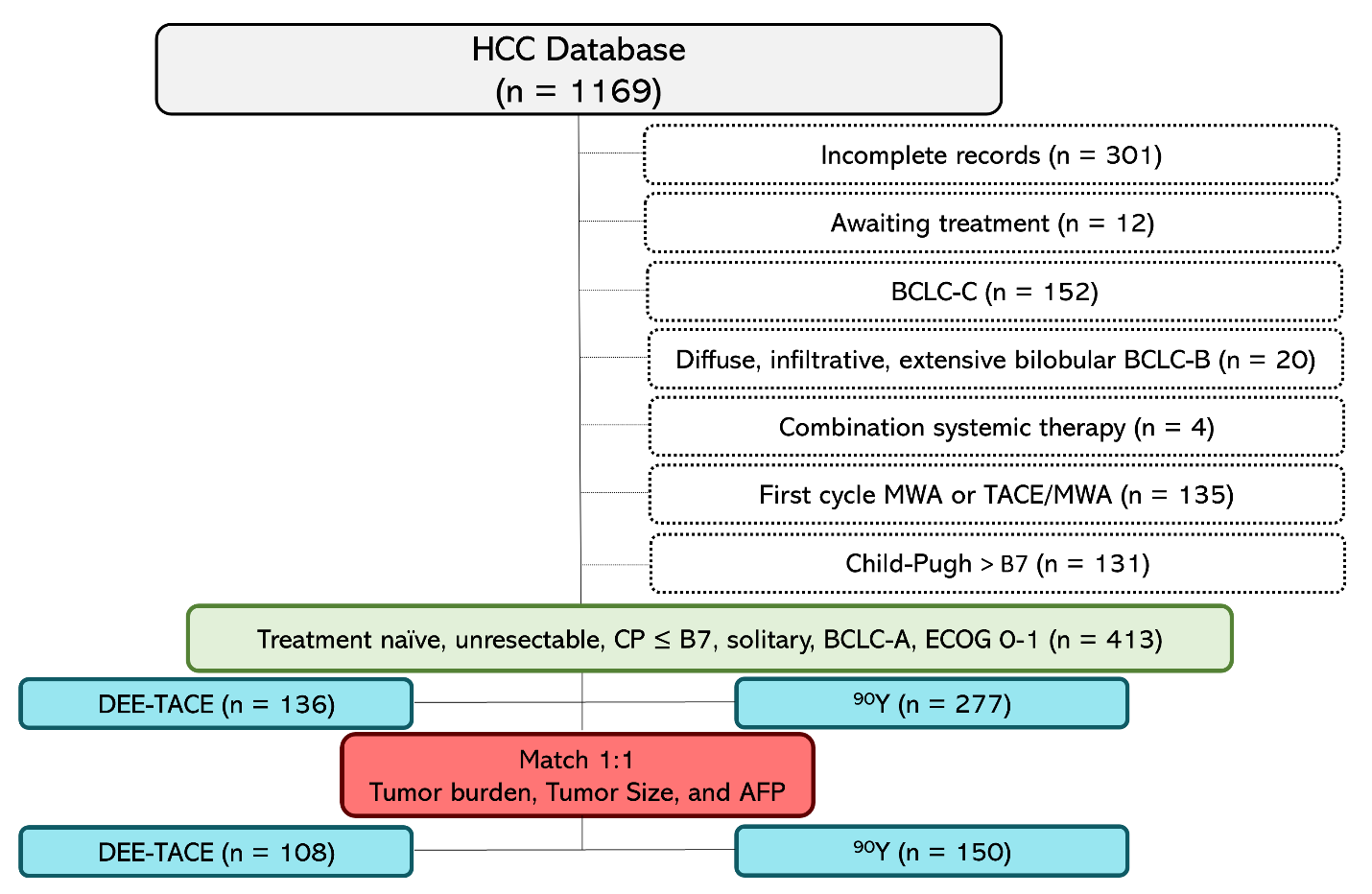
**

**Supplemental Figure 1. Consort Diagram**

**Supplemental Figure 2. Distribution plots Before and After PSM for Categorical Variables.** Plots shown for (A) sex, (B) race, (C) ECOG score, (D) Child-Pugh score, (E) AFP, (F) BCLC stage, (G) mALBI, and (H) multifocal disease in match and unmatched cohort.

**A B**

**C D**

**E F**

**G H** **I J** **K**

**Supplemental Figure 3. Distribution plots Before and After PSM for Continuous Variables.** Plots shown for (A) age, (B) sodium, (C) creatinine, (D) bilirubin, (E) albumin, (F) INR, (G) platelets, (H) MELD score, (I) absolute lymphocyte count, (J) number of tumors, and (K) index lesion size in match and unmatched cohort.

**
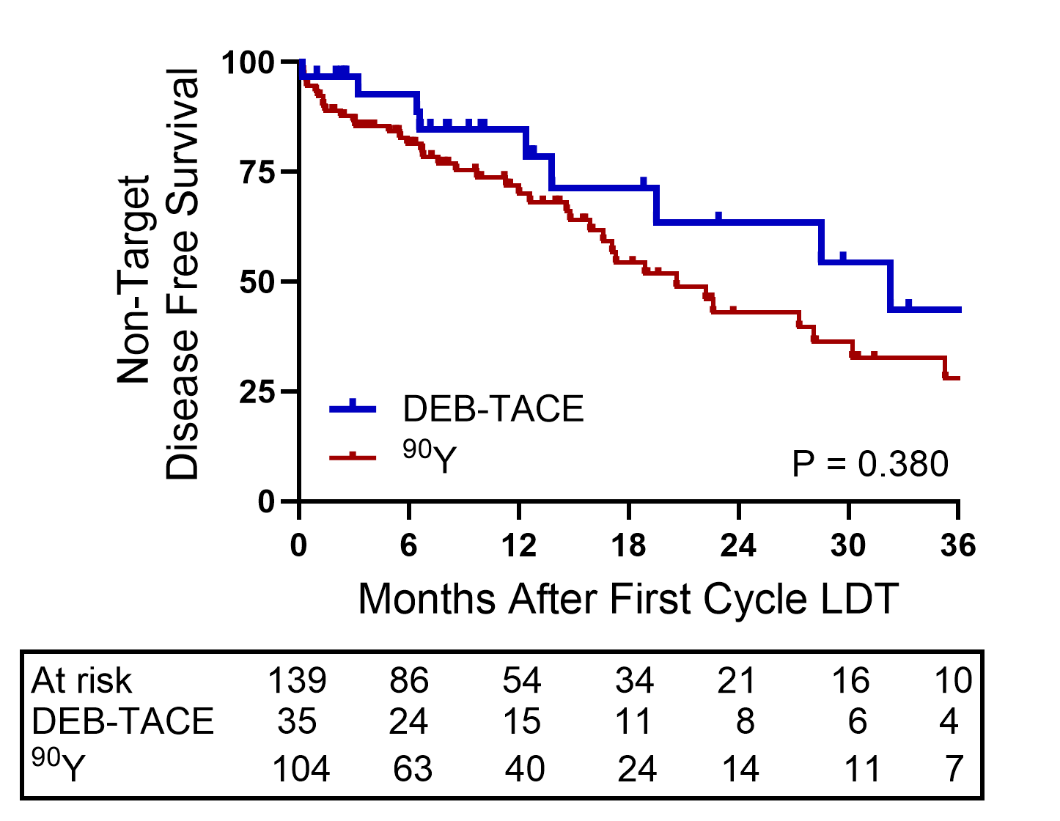
**

**Supplemental Figure 4. Non-Target Disease Free Survival following First Cycle DEB-TACE or ^90^Y.**

**
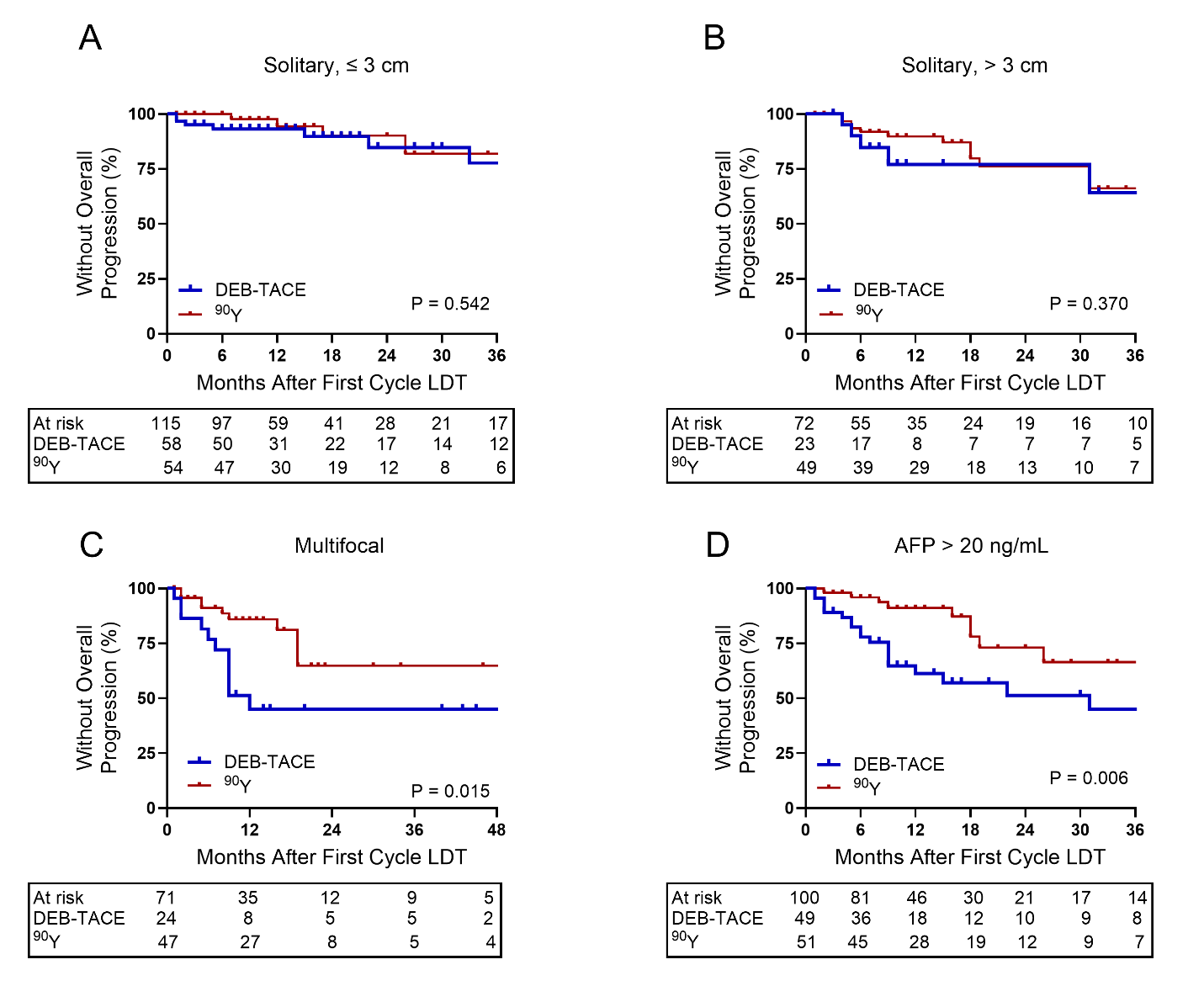
**

**Supplemental Figure 5. Overall Time-to-Progression Rates following First Cycle DEB-TACE or ^90^Y Based on PSM.** Overall TTP following first cycle LDT by modality in patients with (A) solitary lesion ≤ 3cm, (B) solitary lesion > 3 cm, (C) multifocal HCC, and (D) AFP levels > 20 ng/mL at the time of diagnosis.

**
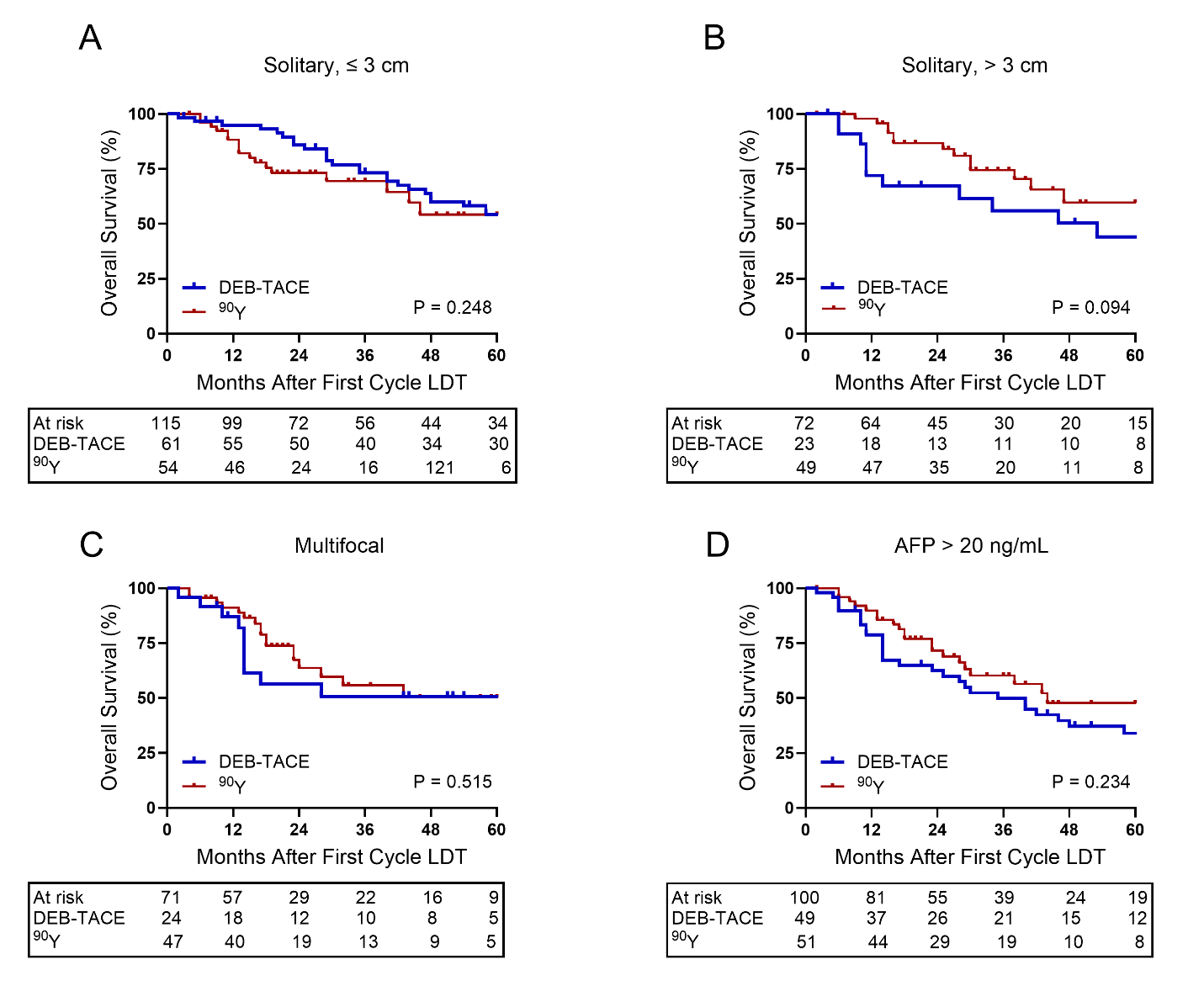
**

**Supplemental Figure 6. Overall Survival following First Cycle DEB-TACE or ^90^Y Based on PSM.** Overall survival following first cycle LDT by modality in patients with (A) solitary lesion ≤ 3cm, (B) solitary lesion > 3 cm, (C) multifocal HCC, and (D) AFP levels > 20 ng/mL at the time of diagnosis.

**SUPPLEMENTAL TABLES**

| **Supplemental Table 1. First Cycle ^90^Y Personalized Dosimetry Characteristics** | |
| --- | --- |
| **^90^Y Treatment Characteristics** |  |
| Perfused volume, mL, median (IQR) | 196 (120-287) |
| Unavailable, n (%) | 14 (9) |
| Dose to volume, Gy, median (IQR) | 468 (365-600) |
| Unavailable, n (%) | 13 (9) |
| Lung shunt fraction, %, median (IQR) | 5.0 (3.3-7.1) |
| Unavailable, n (%) | 2 (1) |
| Abbreviations: Yittrium-90 (^90^Y), Milliliter (mL), Number (No.), Interquartile range (IQR), Gray (Gy). | |

| **Supplemental Table 2. Standardized mean differences before and after matching** | | |
| --- | --- | --- |
|  | **All**  **(N = 419)** | **Matched**  **(N = 258)** |
| **AFP** | -0.038 | -0.044 |
| **Index Size** | 0.397 | 0.068 |
| **Tumor Burden** | 0.264 | 0.207 |
| **Age** | 0.541 | 0.396 |
| **Sex** | -0.099 | -0.172 |
| **Race** |  |  |
| **Black** | 0.066 | 0.208 |
| **White** | 0.001 | -0.120 |
| **Other** | -0.125 | -0.159 |
| **ECOG** | 0.260 | 0.235 |
| **Child-Pugh** |  |  |
| **A5** | -0.361 | -0.289 |
| **A6** | 0.279 | 0.286 |
| **B7** | 0.111 | 0.004 |
| **Sodium** | -0.097 | -0.095 |
| **Creatinine** | 0.077 | 0.054 |
| **Bilirubin** | -0.101 | -0.067 |
| **Albumin** | 0.474 | 0.432 |
| **INR** | -0.374 | -0.261 |
| **Platelets** | 0.243 | 0.193 |
| **MELD 3.0** | -0.104 | -0.109 |
| **Modified ALBI** |  |  |
| **1** | -0.331 | -0.337 |
| **2a** | -0.113 | -0.062 |
| **2b** | 0.323 | 0.301 |
| **3** | 0.125 | 0.066 |
| **ALC** | -0.070 | -0.070 |
| **BCLC Stage** | -0.303 | -0.022 |
| **Tumor Foci** | 0.226 | 0.080 |
| **AFP > 20 ng/mL** | 0.101 | 0.234 |

| **Supplemental Table 3 - Study Cohort Demographics** | | | | |
| --- | --- | --- | --- | --- |
| **Demographic** | **Cohort** | **DEB-TACE** | **^90^Y** | **P-Value** |
| **Patients, n (%)** | 258 | 108 | 150 |  |
| **Treatment date, range** | 1/28/2015 – 4/19/2024 | 1/28/2015 – 12/7/2022 | 6/3/2016 – 4/19/2024 |  |
| **Study follow-up, months, median (IQR)** | 28 (15 – 54) | 44 (14 – 83) | 24 (15 – 41) | **<0.001** |
| **Age at HCC diagnosis, years, median (IQR)** | 65 (61 – 71) | 64 (60 – 69) | 67 (62 – 73) | **<0.001** |
| **Sex, self-reported, male n (%)** | 186 (72) | 73 (68) | 113 (75) | 0.173 |
| **Race, self-reported, n (%)** |  |  |  | 0.154 |
| Caucasian/White | 171 (66) | 68 (63) | 103 (69) |  |
| African American/Black | 72 (28) | 36 (33) | 36 (24) |  |
| Other | 15 (6) | 4 (4) | 11 (7) |  |
| **Cirrhotic etiology, n (%)** |  |  |  | **<0.001** |
| HCV | 131 (51) | 73 (68) | 58 (39) |  |
| MASLD/MASH | 49 (19) | 13 (12) | 36 (24) |  |
| Other | 46 (18) | 13 (12) | 33 (22) |  |
| HCV ALD | 32 (12) | 9 (8) | 23 (15) |  |
| **Scores and Staging** |  |  |  |  |
| **ECOG Performance Status, n (%)** |  |  |  | 0.062 |
| Score 0 | 190 (74) | 73 (68) | 117 (78) |  |
| Score 1 | 68 (26) | 35 (32) | 33 (22) |  |
| **Child-Pugh, n (%)** |  |  |  | 0.053 |
| A5 | 112 (43) | 38 (35) | 74 (49) |  |
| A6 | 108 (42) | 54 (50) | 54 (36) |  |
| B7 | 38 (15) | 16 (15) | 22 (15) |  |
| **Clinical Labs prior to LDT** |  |  |  |  |
| **Sodium, mM, median (IQR)** | 140 (138 – 141) | 139 (138 – 141) | 140 (138 – 141) | 0.827 |
| **Creatinine, mg/dL, median (IQR)** | 0.9 (0.8 – 1.1) | 0.9 (0.8 – 1.1) | 0.9 (0.8 – 1.1) | 0.250 |
| **Bilirubin, mg/dL, median (IQR)** | 0.8 (0.6 – 1.2) | 0.8 (0.6 – 1.2) | 0.8 (0.6 – 1.2) | 0.545 |
| **Albumin, g/dL, median (IQR)** | 3.5 (3.2 – 3.8) | 3.4 (3.1 – 3.7) | 3.6 (3.4 – 3.9) | **0.001** |
| **INR, ratio, median (IQR)** | 1.1 (1.0 – 1.2) | 1.1 (1.0 – 1.2) | 1.1 (1.0 – 1.2) | 0.060 |
| **Platelets, 10^3^/μL, median (IQR)** | 127 (88 – 180) | 113 (81 – 162) | 150 (91 – 186) | **0.025** |
| **MELD 3.0, median (IQR)** | 9 (7 – 11) | 9 (7 – 11) | 9 (7 – 11) | 0.385 |
| **Modified ALBI score, n (%)** |  |  |  | **0.031** |
| Grade 1 | 56 (22) | 15 (14) | 41 (27) |  |
| Grade 2a | 71 (27) | 28 (26) | 43 (29) |  |
| Grade 2b | 121 (47) | 60 (55) | 61 (41) |  |
| Grade 3 | 10 (4) | 5 (5) | 5 (3) |  |
| **ALC, 10^3^/μL, median (IQR)** | 1.5 (1.0 – 2.1) | 1.6 (1.1 – 2.2) | 1.5 (0.9 – 2.0) | 0.139 |
| **HCC Burden and Biomarkers** |  |  |  |  |
| **BCLC Stage, n (%)** |  |  |  | 0.860 |
| A | 221 (86) | 93 (86) | 128 (85) |  |
| B | 37 (14) | 15 (14) | 22 (15) |  |
| **Multifocal, n (%)** |  |  |  | 0.103 |
| Solitary | 187 (72) | 84 (78) | 103 (69) |  |
| Multifocal | 71 (28) | 24 (22) | 47 (31) |  |
| **Index Lesion Diameter, cm, median (IQR)** | 2.9 (2.4 – 3.6) | 2.7 (2.3 – 3.5) | 3.0 (2.4 – 3.7) | 0.107 |
| **AFP, >20 ng/mL, n (%)** |  |  |  | 0.065 |
| Positive | 100 (39) | 49 (45) | 51 (34) |  |
| Negative | 158 (61) | 59 (55) | 99 (66) |  |
| **Abbreviations:** Alpha-fetoprotein (AFP), Absolute Lymphocyte Count (ALC), Alcoholic Liver Disease (ALD), Barcelona Clinic Liver Cancer (BCLC), Drug-Eluting Bead Transarterial Chemoembolization (DEE-TACE), Eastern Cooperative Oncology Group (ECOG), Hepatocellular carcinoma (HCC), Hepatitis C virus (HCV), Interquartile range (IQR), International normalized ratio (INR), Liver-directed therapy (LDT), Metabolic dysfunction-associated steatotic liver disease (MASLD), Metabolic dysfunction-associated steatohepatitis (MASH), Model for End-Stage Liver Disease (MELD), Yttrium-90 (^90^Y). | | | | |

| **Supplemental Table 4. Logistic Regression of Factors Post-PSM and Stage Progression** | | | | |
| --- | --- | --- | --- | --- |
| **Demographic** | **Cohort** | **DEB-TACE** | **^90^Y** | **P-Value** |
| **Age at HCC diagnosis, years, median (IQR)** | 65 (61 – 71) | 64 (60 – 69) | 67 (62 – 73) | 0.374 |
| **Cirrhotic etiology, n (%)** |  |  |  | 0.830 |
| HCV | 131 (51) | 73 (68) | 58 (39) |  |
| MASLD/MASH | 49 (19) | 13 (12) | 36 (24) |  |
| Other | 46 (18) | 13 (12) | 33 (22) |  |
| HCV ALD | 32 (12) | 9 (8) | 23 (15) |  |
| **Albumin, g/dL, median (IQR)** | 3.5 (3.2 – 3.8) | 3.4 (3.1 – 3.7) | 3.6 (3.4 – 3.9) | 0.401 |
| **Modified ALBI score, n (%)** |  |  |  | 0.878 |
| Grade 1 | 56 (22) | 15 (14) | 41 (27) |  |
| Grade 2a | 71 (27) | 28 (26) | 43 (29) |  |
| Grade 2b | 121 (47) | 60 (55) | 61 (41) |  |
| Grade 3 | 10 (4) | 5 (5) | 5 (3) |  |
| **Platelets, 10^3^/μL, median (IQR)** | 127 (88 – 180) | 113 (81 – 162) | 150 (91 – 186) | 0.681 |
| Abbreviations: Propensity score matched (PSM, Alcoholic Liver Disease (ALD), Hepatocellular carcinoma (HCC), Albumin-Bilirubin (ALBI), Doxorubicin-eluting bead transarterial chemoembolization (DEB-TACE), Hepatitis C virus (HCV), Interquartile range (IQR), Metabolic dysfunction-associated steatotic liver disease (MASLD), Metabolic dysfunction-associated steatohepatitis (MASH), Number of patients (n), Yttrium-90 (^90^Y). | | | | |

| **Supplemental Table 5. Target Complete Response Rate** | | | | |
| --- | --- | --- | --- | --- |
| **Demographic** | **Cohort** | **DEB-TACE** | **^90^Y** | **P-Value** |
| **Age at HCC diagnosis, years, median (IQR)** | 65 (61 – 71) | 64 (60 – 69) | 67 (62 – 73) | 0.387 |
| **Cirrhotic etiology, n (%)** |  |  |  | 0.888 |
| HCV | 131 (51) | 73 (68) | 58 (39) |  |
| MASLD/MASH | 49 (19) | 13 (12) | 36 (24) |  |
| Other | 46 (18) | 13 (12) | 33 (22) |  |
| HCV ALD | 32 (12) | 9 (8) | 23 (15) |  |
| **Albumin, g/dL, median (IQR)** | 3.5 (3.2 – 3.8) | 3.4 (3.1 – 3.7) | 3.6 (3.4 – 3.9) | 0.066 |
| **Modified ALBI score, n (%)** |  |  |  |  |
| Grade 1 | 56 (22) | 15 (14) | 41 (27) |  |
| Grade 2a | 71 (27) | 28 (26) | 43 (29) |  |
| Grade 2b | 121 (47) | 60 (55) | 61 (41) |  |
| Grade 3 | 10 (4) | 5 (5) | 5 (3) |  |
| **Platelets, 10^3^/μL, median (IQR)** | 127 (88 – 180) | 113 (81 – 162) | 150 (91 – 186) | 0.834 |
| Abbreviations: Alcoholic Liver Disease (ALD), Hepatocellular carcinoma (HCC), Albumin-Bilirubin (ALBI), Doxorubicin-eluting bead transarterial chemoembolization (DEB-TACE), Hepatitis C virus (HCV), Interquartile range (IQR), Metabolic dysfunction-associated steatotic liver disease (MASLD), Metabolic dysfunction-associated steatohepatitis (MASH), Number of patients (n), Yttrium-90 (90Y). | | | | |

| **Supplemental Table 6. Initial Incomplete Responders Treated to Target Complete Response** | | | |
| --- | --- | --- | --- |
|  | **DEB-TACE** | **^90^Y** | **P value** |
| **No. of Patients with IC response, n, % of total** | 71 (67) | 42 (29) |  |
| **Eventual Target CR, n (% of IC responders)** |  |  | 0.615 |
| Yes | 44 (62) | 28 (67) |  |
| No | 27 (38) | 14 (33) |  |
| **No. of LDT to Target CR, median (IQR)** | 2 (2 – 3) | 2 (2 – 3) | 0.949 |
| **Time until Target CR, months, median (IQR)** | 6 (5 – 9) | 6 (5 – 8) | 0.949 |
| **Single Modality Type** | 24 (57) | 16 (57) |  |
| **Switched Modality** | 18 (43) | 12 (43) |  |
| **No. of Imaging Appts to Target CR, median (IQR)** | 3 (3 – 4) | 3 (3 – 4) | 0.954 |
| **Abbreviations:** Drug-Eluting Bead Transarterial Chemoembolization (DEB-TACE), Yttrium-90 (^90^Y), Incomplete (IC), Complete response (CR), Interquartile range (IQR). | | | |
