# Supplementary material for: Radioembolization (90Y) achieves higher response rates and reduces progression risk compared with DEB-TACE in hepatocellular carcinoma
Source: Hepatol Commun. 2026 Apr 17;10(5):e0935. doi: 10.1097/HC9.0000000000000935 (PMC13090074; doi:10.1097/HC9.0000000000000935)
Supplement: Supplementary file 2 [file hc9-10-e0935-s002.docx]

**SUPPLEMENTAL FIGURES**

**
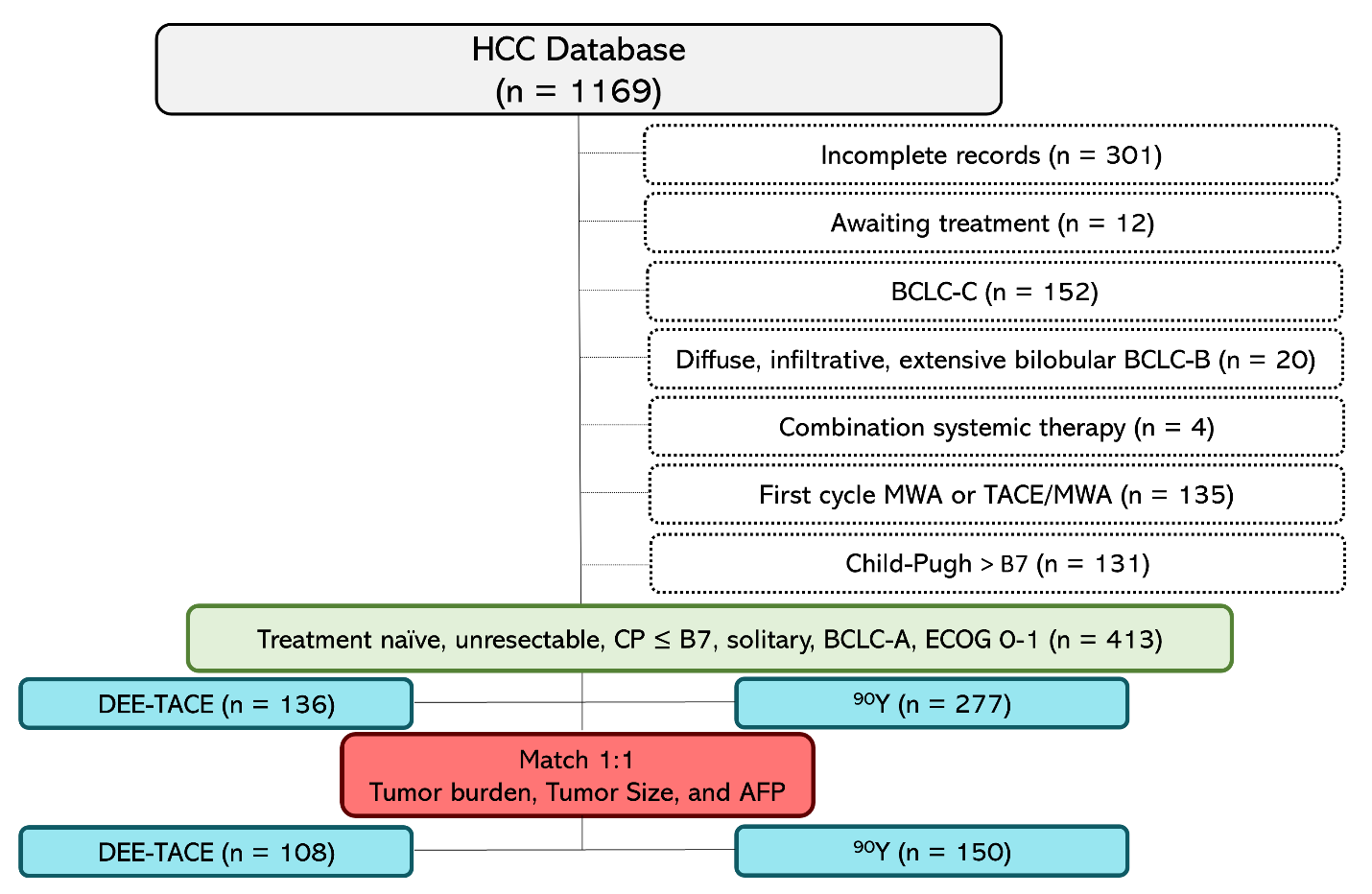
**

**Supplemental Figure 1. Consort Diagram**

**
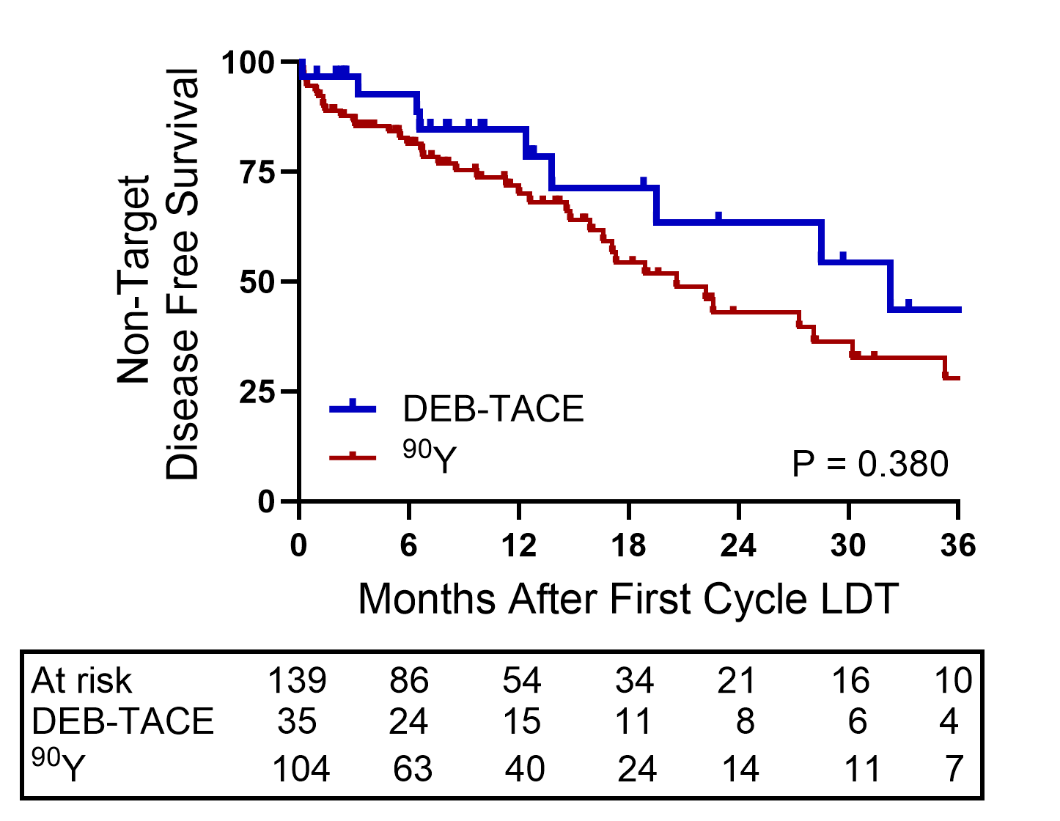
**

**Supplemental Figure 2. Non-Target Disease Free Survival following First Cycle DEB-TACE or ^90^Y.**

**
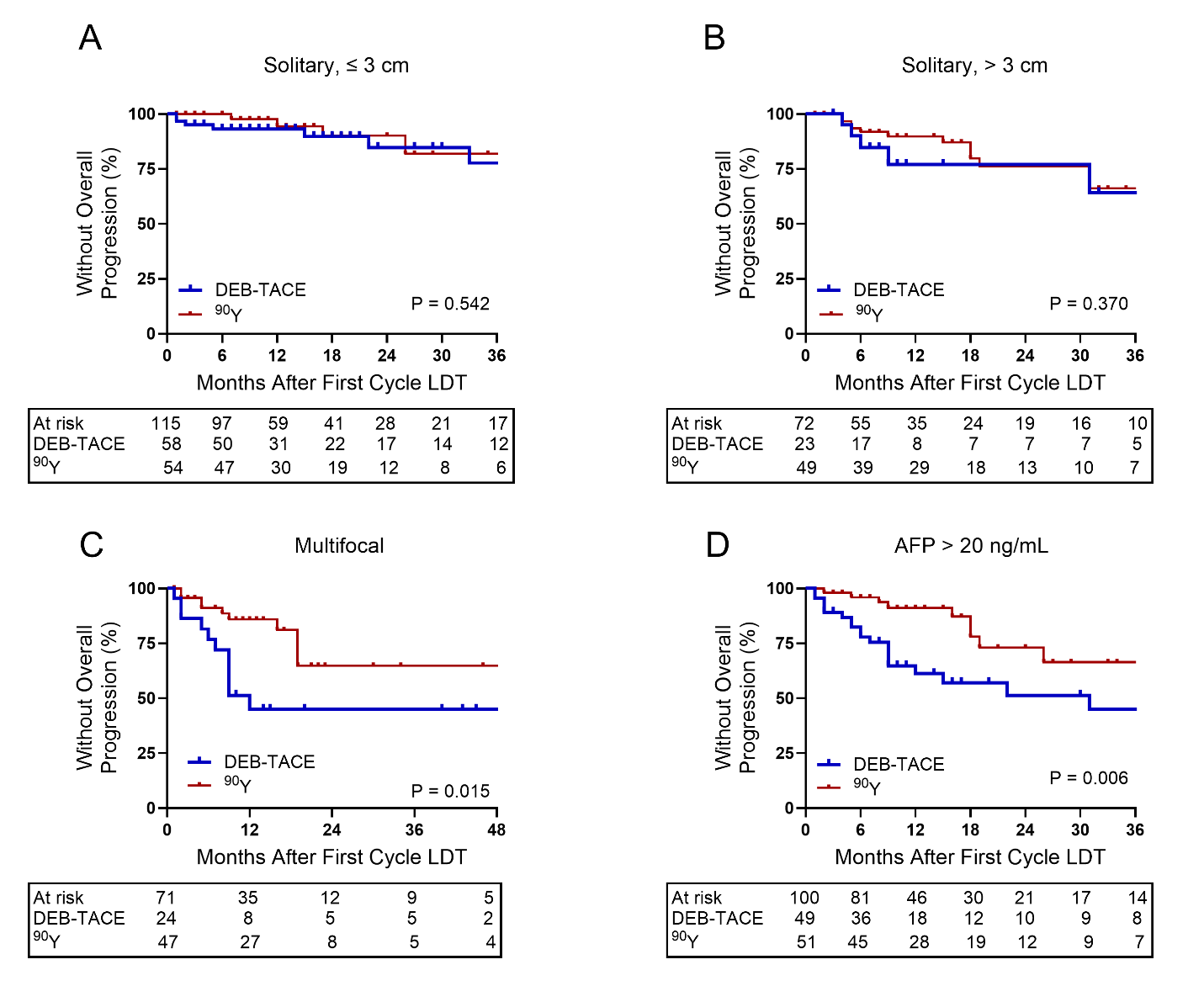
**

**Supplemental Figure 3. Overall Time-to-Progression Rates following First Cycle DEB-TACE or ^90^Y Based on PSM.** Overall TTP following first cycle LDT by modality in patients with (A) solitary lesion ≤ 3cm, (B) solitary lesion > 3 cm, (C) multifocal HCC, and (D) AFP levels > 20 ng/mL at the time of diagnosis.

**
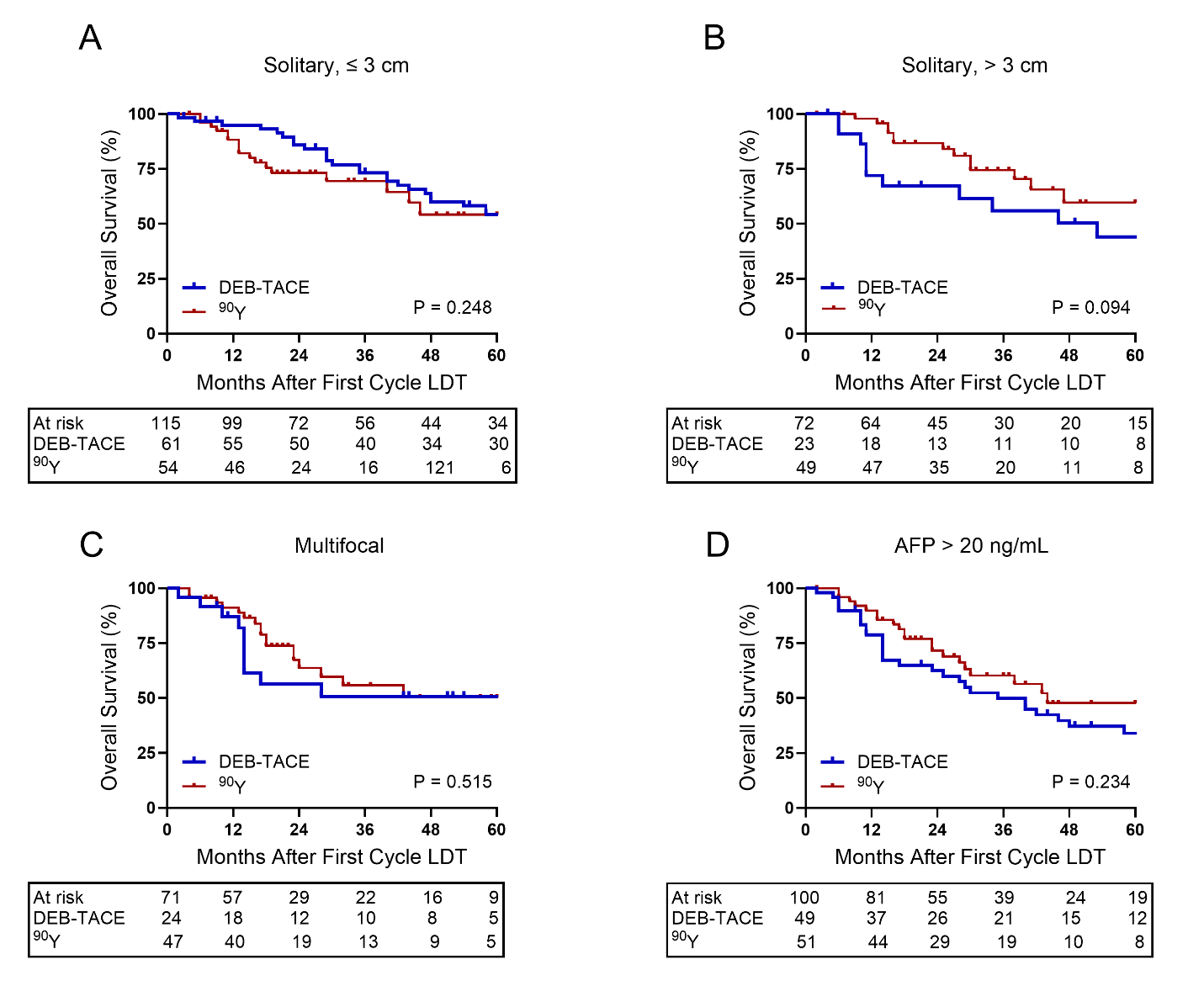
**

**Supplemental Figure 4. Overall Survival following First Cycle DEB-TACE or ^90^Y Based on PSM.** Overall survival following first cycle LDT by modality in patients with (A) solitary lesion ≤ 3cm, (B) solitary lesion > 3 cm, (C) multifocal HCC, and (D) AFP levels > 20 ng/mL at the time of diagnosis.

**SUPPLEMENTAL TABLES**

| **Supplemental Table 1. First Cycle ^90^Y Personalized Dosimetry Characteristics** | |
| --- | --- |
| **^90^Y Treatment Characteristics** |  |
| Perfused volume, mL, median (IQR) | 196 (120-287) |
| Unavailable, n (%) | 14 (9) |
| Dose to volume, Gy, median (IQR) | 468 (365-600) |
| Unavailable, n (%) | 13 (9) |
| Lung shunt fraction, %, median (IQR) | 5.0 (3.3-7.1) |
| Unavailable, n (%) | 2 (1) |
| Abbreviations: Yittrium-90 (^90^Y), Milliliter (mL), Number (No.), Interquartile range (IQR), Gray (Gy). | |

| **Supplemental Table 2 - Study Cohort Demographics** | | | | |
| --- | --- | --- | --- | --- |
| **Demographic** | **Cohort** | **DEB-TACE** | **^90^Y** | **P-Value** |
| **Patients, n (%)** | 258 | 108 | 150 |  |
| **Treatment date, range** | 1/28/2015 – 4/19/2024 | 1/28/2015 – 12/7/2022 | 6/3/2016 – 4/19/2024 |  |
| **Study follow-up, months, median (IQR)** | 28 (15 – 54) | 44 (14 – 83) | 24 (15 – 41) | **<0.001** |
| **Age at HCC diagnosis, years, median (IQR)** | 65 (61 – 71) | 64 (60 – 69) | 67 (62 – 73) | **<0.001** |
| **Sex, self-reported, male n (%)** | 186 (72) | 73 (68) | 113 (75) | 0.173 |
| **Race, self-reported, n (%)** |  |  |  | 0.154 |
| Caucasian/White | 171 (66) | 68 (63) | 103 (69) |  |
| African American/Black | 72 (28) | 36 (33) | 36 (24) |  |
| Other | 15 (6) | 4 (4) | 11 (7) |  |
| **Cirrhotic etiology, n (%)** |  |  |  | **<0.001** |
| HCV | 131 (51) | 73 (68) | 58 (39) |  |
| MASLD/MASH | 49 (19) | 13 (12) | 36 (24) |  |
| Other | 46 (18) | 13 (12) | 33 (22) |  |
| HCV ALD | 32 (12) | 9 (8) | 23 (15) |  |
| **Scores and Staging** |  |  |  |  |
| **ECOG Performance Status, n (%)** |  |  |  | 0.062 |
| Score 0 | 190 (74) | 73 (68) | 117 (78) |  |
| Score 1 | 68 (26) | 35 (32) | 33 (22) |  |
| **Child-Pugh, n (%)** |  |  |  | 0.053 |
| A5 | 112 (43) | 38 (35) | 74 (49) |  |
| A6 | 108 (42) | 54 (50) | 54 (36) |  |
| B7 | 38 (15) | 16 (15) | 22 (15) |  |
| **Clinical Labs prior to LDT** |  |  |  |  |
| **Sodium, mM, median (IQR)** | 140 (138 – 141) | 139 (138 – 141) | 140 (138 – 141) | 0.827 |
| **Creatinine, mg/dL, median (IQR)** | 0.9 (0.8 – 1.1) | 0.9 (0.8 – 1.1) | 0.9 (0.8 – 1.1) | 0.250 |
| **Bilirubin, mg/dL, median (IQR)** | 0.8 (0.6 – 1.2) | 0.8 (0.6 – 1.2) | 0.8 (0.6 – 1.2) | 0.545 |
| **Albumin, g/dL, median (IQR)** | 3.5 (3.2 – 3.8) | 3.4 (3.1 – 3.7) | 3.6 (3.4 – 3.9) | **0.001** |
| **INR, ratio, median (IQR)** | 1.1 (1.0 – 1.2) | 1.1 (1.0 – 1.2) | 1.1 (1.0 – 1.2) | 0.060 |
| **Platelets, 10^3^/μL, median (IQR)** | 127 (88 – 180) | 113 (81 – 162) | 150 (91 – 186) | **0.025** |
| **MELD 3.0, median (IQR)** | 9 (7 – 11) | 9 (7 – 11) | 9 (7 – 11) | 0.385 |
| **Modified ALBI score, n (%)** |  |  |  | **0.031** |
| Grade 1 | 56 (22) | 15 (14) | 41 (27) |  |
| Grade 2a | 71 (27) | 28 (26) | 43 (29) |  |
| Grade 2b | 121 (47) | 60 (55) | 61 (41) |  |
| Grade 3 | 10 (4) | 5 (5) | 5 (3) |  |
| **ALC, 10^3^/μL, median (IQR)** | 1.5 (1.0 – 2.1) | 1.6 (1.1 – 2.2) | 1.5 (0.9 – 2.0) | 0.139 |
| **HCC Burden and Biomarkers** |  |  |  |  |
| **BCLC Stage, n (%)** |  |  |  | 0.860 |
| A | 221 (86) | 93 (86) | 128 (85) |  |
| B | 37 (14) | 15 (14) | 22 (15) |  |
| **Multifocal, n (%)** |  |  |  | 0.103 |
| Solitary | 187 (72) | 84 (78) | 103 (69) |  |
| Multifocal | 71 (28) | 24 (22) | 47 (31) |  |
| **Index Lesion Diameter, cm, median (IQR)** | 2.9 (2.4 – 3.6) | 2.7 (2.3 – 3.5) | 3.0 (2.4 – 3.7) | 0.107 |
| **AFP, >20 ng/mL, n (%)** |  |  |  | 0.065 |
| Positive | 100 (39) | 49 (45) | 51 (34) |  |
| Negative | 158 (61) | 59 (55) | 99 (66) |  |
| **Abbreviations:** Alpha-fetoprotein (AFP), Absolute Lymphocyte Count (ALC), Alcoholic Liver Disease (ALD), Barcelona Clinic Liver Cancer (BCLC), Drug-Eluting Bead Transarterial Chemoembolization (DEE-TACE), Eastern Cooperative Oncology Group (ECOG), Hepatocellular carcinoma (HCC), Hepatitis C virus (HCV), Interquartile range (IQR), International normalized ratio (INR), Liver-directed therapy (LDT), Metabolic dysfunction-associated steatotic liver disease (MASLD), Metabolic dysfunction-associated steatohepatitis (MASH), Model for End-Stage Liver Disease (MELD), Yttrium-90 (^90^Y). | | | | |

| **Supplemental Table 3. Logistic Regression of Factors Post-PSM and Stage Progression** | | | | |
| --- | --- | --- | --- | --- |
| **Demographic** | **Cohort** | **DEB-TACE** | **^90^Y** | **P-Value** |
| **Age at HCC diagnosis, years, median (IQR)** | 65 (61 – 71) | 64 (60 – 69) | 67 (62 – 73) | 0.374 |
| **Cirrhotic etiology, n (%)** |  |  |  | 0.830 |
| HCV | 131 (51) | 73 (68) | 58 (39) |  |
| MASLD/MASH | 49 (19) | 13 (12) | 36 (24) |  |
| Other | 46 (18) | 13 (12) | 33 (22) |  |
| HCV ALD | 32 (12) | 9 (8) | 23 (15) |  |
| **Albumin, g/dL, median (IQR)** | 3.5 (3.2 – 3.8) | 3.4 (3.1 – 3.7) | 3.6 (3.4 – 3.9) | 0.401 |
| **Modified ALBI score, n (%)** |  |  |  | 0.878 |
| Grade 1 | 56 (22) | 15 (14) | 41 (27) |  |
| Grade 2a | 71 (27) | 28 (26) | 43 (29) |  |
| Grade 2b | 121 (47) | 60 (55) | 61 (41) |  |
| Grade 3 | 10 (4) | 5 (5) | 5 (3) |  |
| **Platelets, 10^3^/μL, median (IQR)** | 127 (88 – 180) | 113 (81 – 162) | 150 (91 – 186) | 0.681 |
| Abbreviations: Propensity score matched (PSM, Alcoholic Liver Disease (ALD), Hepatocellular carcinoma (HCC), Albumin-Bilirubin (ALBI), Doxorubicin-eluting bead transarterial chemoembolization (DEB-TACE), Hepatitis C virus (HCV), Interquartile range (IQR), Metabolic dysfunction-associated steatotic liver disease (MASLD), Metabolic dysfunction-associated steatohepatitis (MASH), Number of patients (n), Yttrium-90 (^90^Y). | | | | |

| **Supplemental Table 4. Target Complete Response Rate** | | | | |
| --- | --- | --- | --- | --- |
| **Demographic** | **Cohort** | **DEB-TACE** | **^90^Y** | **P-Value** |
| **Age at HCC diagnosis, years, median (IQR)** | 65 (61 – 71) | 64 (60 – 69) | 67 (62 – 73) | 0.387 |
| **Cirrhotic etiology, n (%)** |  |  |  | 0.888 |
| HCV | 131 (51) | 73 (68) | 58 (39) |  |
| MASLD/MASH | 49 (19) | 13 (12) | 36 (24) |  |
| Other | 46 (18) | 13 (12) | 33 (22) |  |
| HCV ALD | 32 (12) | 9 (8) | 23 (15) |  |
| **Albumin, g/dL, median (IQR)** | 3.5 (3.2 – 3.8) | 3.4 (3.1 – 3.7) | 3.6 (3.4 – 3.9) | 0.066 |
| **Modified ALBI score, n (%)** |  |  |  |  |
| Grade 1 | 56 (22) | 15 (14) | 41 (27) |  |
| Grade 2a | 71 (27) | 28 (26) | 43 (29) |  |
| Grade 2b | 121 (47) | 60 (55) | 61 (41) |  |
| Grade 3 | 10 (4) | 5 (5) | 5 (3) |  |
| **Platelets, 10^3^/μL, median (IQR)** | 127 (88 – 180) | 113 (81 – 162) | 150 (91 – 186) | 0.834 |
| Abbreviations: Alcoholic Liver Disease (ALD), Hepatocellular carcinoma (HCC), Albumin-Bilirubin (ALBI), Doxorubicin-eluting bead transarterial chemoembolization (DEB-TACE), Hepatitis C virus (HCV), Interquartile range (IQR), Metabolic dysfunction-associated steatotic liver disease (MASLD), Metabolic dysfunction-associated steatohepatitis (MASH), Number of patients (n), Yttrium-90 (90Y). | | | | |
